# Deep Learning for Cervical Spine Radiography: Automated Measurement of Intervertebral and Neural Foraminal Distances

**DOI:** 10.3390/diagnostics15172162

**Published:** 2025-08-26

**Authors:** Ya-Yun Huang, Hong-Kai Wang, Tsun-Kuang Chi, Chao-Shin Liu, Sung-Hsin Tsai, Sze-Teng Liong, Tsung-Yi Chen, Kuo-Chen Li, Wei-Chen Tu, Patricia Angela R. Abu

**Affiliations:** 1 Program on Semiconductor Manufacturing Technology, Academy of Innovative Semiconductor and Sustainable Manufacturing, National Cheng Kung University, Tainan City 701401, Taiwan; m28124023@gs.ncku.edu.tw (Y.-Y.H.); wctu@gs.ncku.edu.tw (W.-C.T.); 2 Department of Neurosurgery, Linkou Chang Gung Memorial Hospital, Taoyuan City 333423, Taiwan; cakekevin3211@hotmail.com; 3 Department of Electrical Engineering, Ming Chi University of Technology, New Taipei City 243303, Taiwan; 4 Department of Electronic Engineering, Feng Chia University, Taichung City 40724, Taiwan; m1302521@o365.fcu.edu.tw (C.-S.L.); stliong@fcu.edu.tw (S.-T.L.); 5 Department of Medical Education, Chang Gung Memorial Hospital Linkou, Taoyuan City 333423, Taiwan; mpq689@cgmh.org.tw; 6 Department of Information Management, Chung Yuan Christian University, Taoyuan City 320317, Taiwan; kuochen@cycu.edu.tw; 7 Department of Electronic Engineering, National Cheng Kung University, Tainan City 701401, Taiwan; 8 Ateneo Laboratory for Intelligent Visual Environments, Department of Information Systems and Computer Science, Ateneo de Manila University, Quezon City 1108, Philippines; pabu@ateneo.edu

**Keywords:** YOLOv8, cervical spine localization, neural foramen distance measurement, deep learning, vertebral segmentation, spinal radiography

## Abstract

**Background/Objectives:** The precise localization of cervical vertebrae in X-ray imaging was essential for effective diagnosis and treatment planning, particularly as the prevalence of cervical degenerative conditions increased with an aging population. Vertebrae from C2 to C7 were commonly affected by disorders such as ossification of the posterior longitudinal ligament (OPLL) and nerve compression caused by posterior osteophytes, necessitating thorough evaluation. However, manual annotation remained a major aspect of traditional clinical procedures, making it challenging to manage increasing patient volumes and large-scale medical imaging data. **Methods:** To address this issue, this study presented an automated approach for localizing cervical vertebrae and measuring neural foraminal distance. The proposed technique analyzed the neural foramen distance and intervertebral space using image enhancement to determine the degree of nerve compression. YOLOv8 was employed to detect and segment the cervical vertebrae. Moreover, by integrating automated cervical spine analysis with advanced imaging technologies, the system enabled rapid detection of abnormal intervertebral disc gaps, facilitating early identification of degenerative changes. **Results:** According to the results, the system achieved a spine localization accuracy of 99.5%, representing an 11.7% improvement over existing approaches. Notably, it outperformed previous methods by 66.67% in recognizing the C7 vertebra, achieving a perfect 100% accuracy. **Conclusions:** Furthermore, the system significantly streamlined the diagnostic workflow by processing each X-ray image in just 17.9 milliseconds. This approach markedly improved overall diagnostic efficiency.

## 1. Introduction

As the global population continues to grow, the increasing demand for medical services has become one of the most pressing challenges in modern healthcare. To alleviate the workload of healthcare professionals and enhance clinical efficiency, interdisciplinary collaboration between medicine and technology has become increasingly essential. Artificial intelligence (AI) has significantly advanced the automation of medical diagnostics and assisted in early-stage evaluations, thereby improving the overall efficiency of medical consultations [1]. In particular, deep learning techniques [2] play a vital role in computer-aided diagnosis (CAD), especially in the field of neurosurgery. AI models such as YOLO and Faster R-CNN have been widely adopted for the detection and localization of cervical and spinal diseases [3,4,5,6], enabling accurate vertebrae localization and significantly advancing diagnostic precision in clinical practice.

Computed Tomography (CT), Magnetic Resonance Imaging (MRI), and X-ray imaging are commonly employed diagnostic tools for cervical spine disorders [7,8]. Among them, X-ray imaging offers several advantages, including fast acquisition, low cost, and minimal radiation exposure [9,10]. Despite its simplicity, the X-ray provides essential structural information such as vertebral bodies, intervertebral discs, and neural foramina, supporting the diagnosis of cervical conditions including fractures, disc herniation, the ossification of the posterior longitudinal ligament (OPLL), and foraminal stenosis [11,12,13]. Due to these characteristics, the X-ray remains a critical tool for initial assessment and disease progression monitoring, enabling timely diagnosis and treatment planning that can ultimately improve clinical efficiency and patient outcomes.

In cervical spine imaging analysis, the intervertebral distance [14] serves as a crucial quantitative marker for evaluating intervertebral disc degeneration, spinal stability, and spinal stenosis. Accurate measurement of this distance plays a key role in diagnosing cervical spine disorders and assessing the severity of structural abnormalities. A reduction in intervertebral spacing is commonly associated with degenerative disc disease or disc herniation, which can alter the biomechanical load distribution along the spine, potentially resulting in chronic pain, limited mobility, or neurological symptoms. Additionally, anterior–posterior vertebral distance measurements allow clinicians to assess the impact of structural changes such as bone spurs or OPLL on surrounding neural elements. A significantly narrowed distance may indicate spinal cord compression, which can lead to neurological deficits, including limb weakness, sensory disturbances, or chronic discomfort, further impairing a patient’s quality of life.

**Figure 1:**
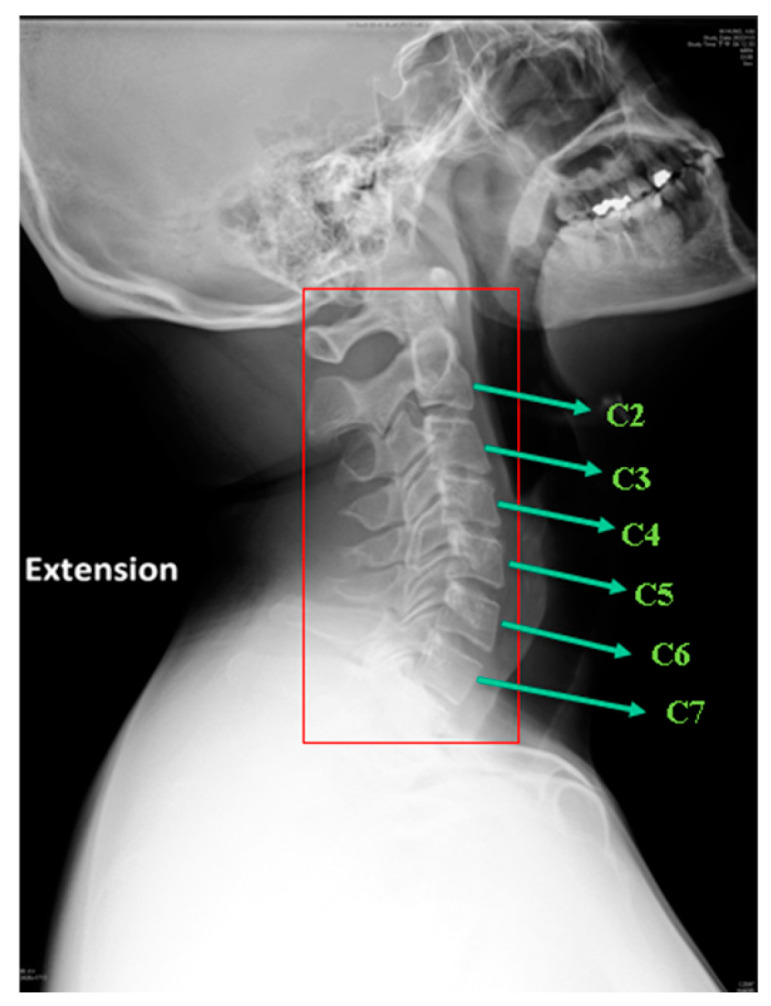
The cervical spine radiography with cervical vertebrae labels.

While X-ray imaging remains essential for cervical spine assessment, existing X-ray-based localization methods predominantly focus on the C3 to C7 vertebrae, as shown in Figure 1, and still encounter significant challenges, particularly in accurately identifying the C7 and C2 vertebrae. Precise localization of C7 is often hindered by low image contrast and potential shoulder overlap, which limits the effectiveness of traditional techniques. Furthermore, identifying the C2 vertebra (axis) poses an even greater difficulty due to its close anatomical connection with C1 (atlas) and its unique morphological characteristics, making accurate detection nearly impossible using conventional methods.

To address these limitations, this study proposes an automated system for measuring intervertebral distances and vertebra-to-neural foramen distances in cervical spine radiographs (CSRs). The system operates in two stages. First, it localizes cervical vertebrae and neural foramina by using YOLOv8 [15,16] to automatically identify regions of interest and by training two dedicated models for vertebra and foramen localization. Second, once the vertebrae and neural foramina have been localized, it computes intervertebral and vertebra-to-neural foramen distances. Beyond achieving high accuracy, the proposed approach markedly streamlines the localization and measurement workflow, reducing processing time and the need for manual annotation. In contrast to traditional annotation-heavy methods, the system enables automated, efficient CSR analysis and distance calculation, thereby improving clinicians’ annotation throughput and diagnostic efficiency and allowing greater focus on treatment planning. Overall, this study contributes to the advancement of automated cervical spine analysis, offering a reliable and practical tool to support clinical decision making.

## 2. Method

**Figure 2:**
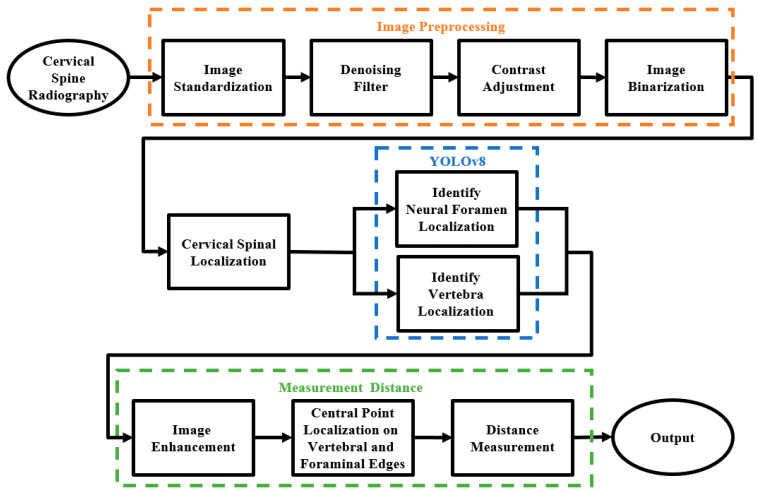
Flow chart of this study.

The flowchart of the overall system proposed in this study was shown in Figure 2. The cervical spine region was first located and cropped to ensure accurate analysis. The YOLOv8 model was then employed to simultaneously detect and label vertebrae from C2 to C7 and neural foramina, while also measuring the distance between each vertebra and its corresponding neural foramen. These measurements provided critical information for evaluating degenerative changes and potential nerve compression. The identified vertebrae served as references for further analysis, in which intervertebral distances were precisely calculated using image enhancement and coordinate transformation.

### 2.1. Image Preprocessing

In this study, efficient image preprocessing was critical to enhancing the overall system accuracy [17,18]. The primary goal of this stage was to accurately extract the cervical spine region from CSR images. To address the issue of grayscale non-uniformity commonly observed in X-ray imaging, various preprocessing methods were applied to improve the contrast between the vertebrae and the background. This step included image standardization, noise reduction, contrast enhancement, and image binarization. Together, these processes improved the visibility of cervical spine contours and supported more reliable downstream analysis.

**Figure 3:**
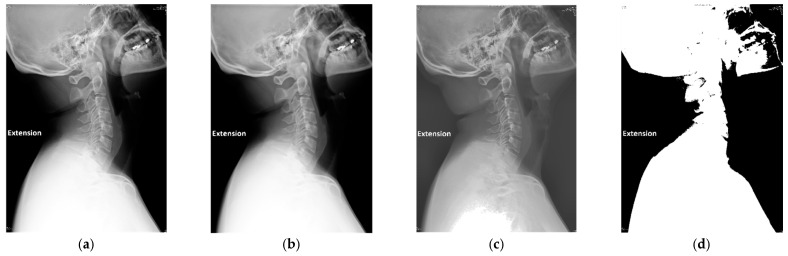
The result for the cervical spine radiography by different preprocessing. (**a**) Original image. (**b**) The median filtered image. (**c**) The image after contrast enhancement. (**d**) Binarized image.

To ensure data consistency and compatibility with the deep learning model, all X-ray images were resized to 512 × 512 pixels. In addition, this study applied a median filter for noise reduction, which was particularly effective against salt-and-pepper noise commonly found in medical imaging [19,20,21]. The median filter was a widely used image processing technique that smoothed the image while preserving important edge details, making it well-suited for denoising cervical spine images without compromising anatomical structures. Unlike linear filters, each pixel was updated with the median value derived from its neighboring pixels when using a median filter, thereby avoiding excessive blurring and maintaining the clarity of vertebral boundaries. This process effectively preserved the fine details of cervical joints and their edges, which were critical for accurate localization. The mathematical formulation of the median filter was presented in Equation (1), and its denoising effect was shown in Figure 3b.

(1) $$g_{\left(x,\;y\right)}=\mathrm{median}\left\{f_{\left(i,\;j\right)}|\left(i,j\right)\;\in {\;S}_{\left(x,\;y\right)}\right\}$$

To further enhance the visibility of cervical vertebrae structures, this study integrated two contrast enhancement methods: Histogram Equalization (HE) and Contrast-Limited Adaptive Histogram Equalization (CLAHE) [22,23]. HE improved global contrast by redistributing grayscale values across the entire image histogram, while CLAHE enhanced local contrast adaptively within small regions and prevented over-amplification of noise by limiting contrast enhancement. The combination of these methods effectively improved both global and local contrast, resulting in clearer delineation of vertebral boundaries and anatomical features. The enhancement results were shown in Figure 3c.

Following contrast enhancement, adaptive thresholding was applied to detect variations in pixel intensity, which supported cervical spine localization and ensured stable separation between the cervical spine and the background under varying exposure conditions. To further refine this separation, Otsu’s thresholding method [24] was employed to isolate the cervical spine from the background. This method, particularly effective for images with bimodal intensity distributions, calculated the optimal threshold by maximizing inter-class variance, as described in Equation (2). This step significantly improved segmentation accuracy and the visibility of cervical vertebrae, as shown in Figure 3d.

(2) $$\sigma^{2}\omega ={\omega_{0}\sigma_{0}}^{2}+{\omega_{1}\sigma_{1}}^{2}$$

### 2.2. Cervical Spine Localization

**Figure 4:**
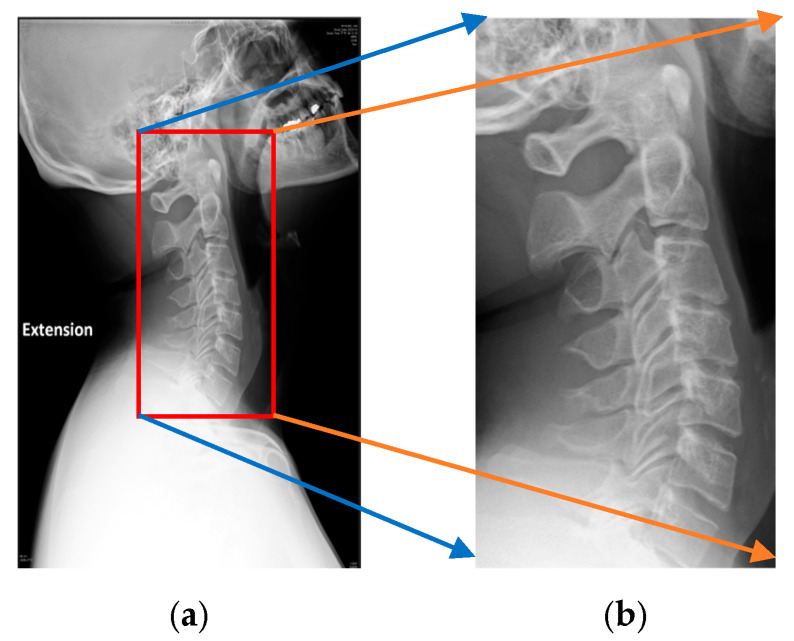
The result of the X-ray cervical spine localization. (**a**) Original cervical spine radiography. (**b**) The image after algorithm processing.

Following the image preprocessing step, the algorithm processed the binarized images by scanning each row to identify the one with the fewest white pixels, typically corresponding to the area of lowest pixel density. This row served as a key reference for determining the lateral boundaries of the cervical spine. To locate the central X-coordinate, the study detected the first and last transition points from black to white along the identified row. It then expanded leftward and rightward until the pixel values returned to black, thereby defining the full width of the cervical spine. To ensure complete coverage and avoid loss of anatomical information, a padding of 50 pixels was added to both sides. A similar approach was applied along the Y-axis: vertical transitions were analyzed at the leftmost and rightmost X-boundaries to determine the superior and inferior edges of the spine. Additional padding was added to the upper and lower margins to retain essential contextual information. This cropping step eliminated irrelevant information, ensured consistent extraction across all images, and enhanced the reliability of subsequent vertebrae recognition. The results of the cervical spine localization were shown in Figure 4.

### 2.3. Vertebra and Neural Foramen Localization by Yolov8s

To ensure clinical applicability, this study was conducted in collaboration with two board-certified neurosurgeons, each with over three years of clinical experience. The study was approved by the Institutional Review Board (IRB) under approval number 202401261B0. The dataset comprised 200 cervical spine X-ray images collected from Chang Gung Memorial Hospital during the study period, encompassing vertebrae from C2 to C7. All images were clinical studies from adults aged eighteen years or older, and the ratio of males to females was approximately three to one.

At this stage, a vertebra localization model was developed to accurately extract the region of interest (ROI) for subsequent analysis [25,26,27]. This study adopted YOLO, a deep learning-based object detection framework, to enable accurate and efficient real-time localization of cervical vertebrae [28]. Traditional cervical spine identification methods typically involved multiple image processing steps, rendering them unsuitable for real-time applications. In contrast, YOLO performed both object localization and classification simultaneously within a single inference pass, making it particularly well-suited for remote healthcare settings and clinical environments where rapid diagnostic support was essential [29,30].

After comprehensive evaluation, YOLOv8s was selected as the vertebra localization model in this study. Table 1 summarizes the hardware and software platforms used for training the deep learning model in this work. Additionally, a total of 200 CSR images were used to train the YOLOv8s model in this study. To ensure representative sampling and reduce selection bias samples, the dataset randomly split into 160 training, 20 validation, and 20 testing samples, as summarized in Table 2.

### 2.4. Automated Measurement Distance

This study proposed an automated system for measuring intervertebral distances, with standardized outputs serving as valuable references for clinical diagnosis. To ensure accurate and reliable distance computation, the system integrated image enhancement, edge detection, and coordinated transformation method.

To ensure high image quality and preserve the clarity and integrity of vertebral structures, multiple image enhancement techniques were applied, particularly because raw X-ray images often contained irrelevant regions such as soft tissues or imaging artifacts. First, contrast stretching was used to enhance fine details and improve the visibility of vertebral structures under low-contrast conditions. Next, a Gaussian blur filter was applied to smooth the image and reduce edge artifacts that could interfere with further analysis. The image was then binarized, effectively separating the vertebral region from the background and preserving the continuity of the skeletal structures. To further emphasize vertebral boundaries and eliminate small-scale noise, morphological operations were employed. Finally, to ensure that only the vertebral region was retained, the largest connected component was identified and extracted, removing residual non-target regions.

**Figure 5:**
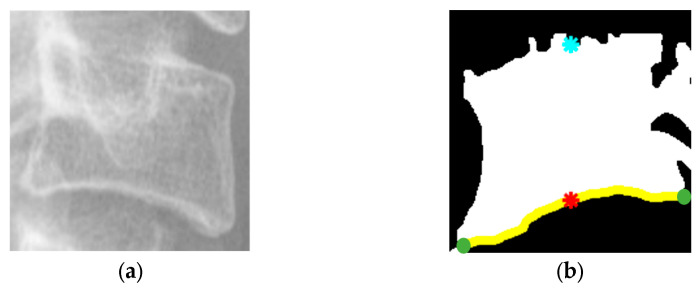
Vertebral image enhancement and central point localization. (**a**) Vertebrae after YOLO-based cropping. (**b**) Image after preprocessing and central point localization.

After image enhancement, the study localized central points separately along the vertebral boundary and along the lateral boundary of the neural foramen. Using the vertebra in Figure 5b as an example, within the largest white contour the bottom-left and bottom-right extreme points were first identified, shown as green dots in Figure 5b. These two points were then used to compute the center along the x-axis. The intersection of this x-axis with the lower white boundary was taken as the lower reference point of the vertebra, shown as a red dot in Figure 5b. Applying the same procedure to the upper boundary yielded the upper reference point of the vertebra, indicated by a blue dot in Figure 5b.

**Figure 6:**
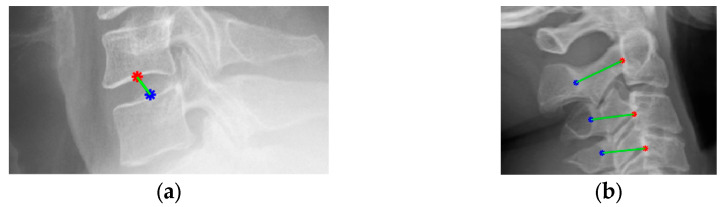
The sample of distance calculation. (**a**) The intervertebral distance. (**b**) The distance between a vertebra and the neural foramen.

Following central-point localization along the vertebral boundary and the lateral border of the neural foramen, the Euclidean distance formula was applied to compute the geometric distance between the two points, as shown in Equation (3). A schematic diagram illustrating the distance calculation was presented in Figure 6.

(3) $$d=\sqrt{{\left(a_{2}-a_{1}\right)}^{2}-{\left(b_{2}-b_{1}\right)}^{2}}$$

## 3. Results

For clarity and completeness, the performance evaluation was discussed in three distinct parts: cervical spine cropping, the module for vertebra localization, and intervertebral distance measurement. Performance evaluation was conducted using Accuracy, Precision, Recall, and Mean Average Precision (mAP) metrics to ensure objectivity and consistency, with their definitions provided in Equations (4)–(8). In this context, true positive (Tp) and true negative (Tn) represented correctly predicted positive and negative samples, respectively, while false positive (Fp) and false negative (Fn) denoted incorrectly predicted positive and negative samples.

(4) $$Accuracy=\frac{Tp+Tn}{Tp+Fp+Tn+Fn}$$

(5) $$Precision=\frac{Tp}{Tp+Fp}$$

(6) $$Recall=\frac{Tp}{Tp+Fn}$$

(7) $$AP=\int_{0}^{1}{Precision}_{\left(\mathrm{Recall}\right)}d\mathrm{Recall}$$

(8) $$mAP=\frac{1}{n}\sum_{i=0}^{n}{AP}_{i}$$

### 3.1. The Performance for Cervical Spine Localization

Compared to using the original input images, the accuracy of vertebral localization was greatly increased by applying cropping based on the cervical spine algorithm. As demonstrated in Table 3, the cropped images successfully eliminated extra background and noise, improving the model’s capacity to identify cervical spine structures. By increasing accuracy from 93.30% to 99.50%, this method highlighted the significance of cervical spine localization techniques in enhancing model precision, especially for difficult vertebrae like C7. Notably, the localization accuracy for the challenging C7 vertebra increased from 87.00% to 100.00%, marking a substantial and impressive improvement.

### 3.2. The Performance for Model Analysis

Through K-fold cross-validation, the proposed system achieved an average accuracy of 98.00% in vertebra localization, with recall and mAP50 reaching 97.46% and 98.60%, respectively, as shown in Table 4. In neural foramen detection, the model also attained excellent performance, achieving precision, recall, and mAP50 values all exceeding 95.50%, as presented in Table 5. These results confirmed the high reliability and detection accuracy of the model, further validating the system’s stability and generalization capability across different data subsets.

In addition, the vertebra localization performance of the proposed method was compared with existing approaches, as summarized in Table 6. The results indicated that the proposed system significantly outperformed both traditional methods [31,32] and more recent studies [33,34] in terms of localization accuracy. This advantage was particularly evident in challenging cases such as the C7 vertebra. Unlike previous methods that often struggled with complex vertebral structures, the proposed approach achieved 100.00% localization accuracy for C7, representing a notable improvement of at least 10.74% over existing methods. Overall, the proposed system maintained an average localization accuracy of 99.50% across all vertebrae, confirming its stable performance and high precision in both routine and complex localization scenarios.

**Table 6:** Comparative analysis of vertebra localization accuracy across different studies.

	Method in [31]	Method in [32]	Method in [33]	Method in [34]	This Work
Over All	93.76%	89.00%	64.50	91.63	99.50%
C2	N/A	N/A	77.50	91.70	99.30%
C3	96.74%	95.00%	33.33	92.20	99.30%
C4	96.65%	97.50%	63.33	92.30	99.40%
C5	95.51%	95.00%	63.33	91.60	99.70%
C6	95.33%	97.50%	85.00	91.70	99.60%
C7	84.55%	60.00%	N/A	90.30	100.00%

**Figure 7:**
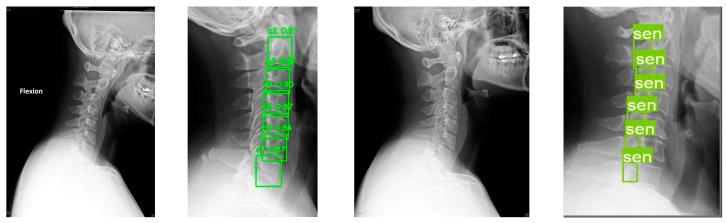
The example of vertebral and neural foraminal localization in this study.

An example of the output generated by the proposed system was shown in Figure 7. It showed the automated localization and labeling of vertebrae and neural foramina using the developed system. In the result, each vertebra from C2 to C7 was labeled along with the corresponding confidence scores. For instance, the model assigned a confidence score of 0.91 to C2 and 0.87 to C7, providing clinicians with a visual reference and an indication of the model’s prediction certainty.

### 3.3. Measurement Distance Analysis

**Figure 8:**
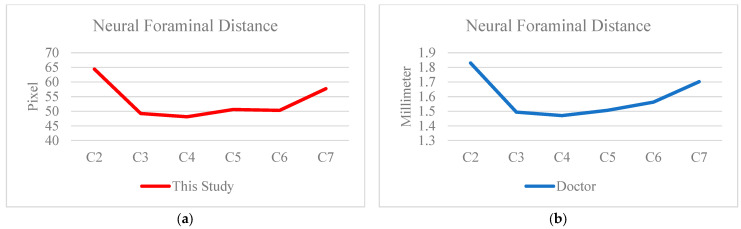
The result of distance measurement for each vertebral and neural foraminal. (**a**) This study. (**b**) Doctor.

**Figure 9:**
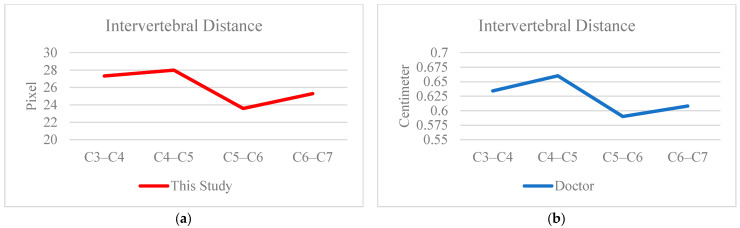
The result of distance measurement for each vertebra. (**a**) This study. (**b**) Doctor.

In the measurement distance analysis section, the distances computed by the proposed system were compared with those manually annotated by doctors. As shown in Figure 8 and Figure 9, a strong visual similarity in linear trends was observed between the two sets of measurements. To objectively validate this trend, five data cases were tested, and the Pearson product-moment correlation coefficient (PPMCC) was calculated. The results, presented in Table 7, showed a high degree of correlation, exceeding 90% for both intervertebral distances and vertebra-to-neural foramen distances. Notably, the correlation for intervertebral distance measurement reached as high as 97.5%, confirming the high reliability and accuracy of the proposed system’s distance computation.

## 4. Discussion

This study proposed an efficient and highly accurate system for the automatic detection and localization of vertebrae and neural structures in CSR images based on the YOLOv8s model. The system successfully identified and labeled the C2 to C7 vertebrae and corresponding neural structures. The proposed method achieved an overall localization accuracy of 99.50%, markedly outperforming prior approaches. Notably, this study achieved a major breakthrough in the localization of the typically challenging C7 vertebra, attaining 100.00% accuracy.

Compared with existing methods, the approaches in [31,32] relied on preprocessing to enhance vertebral contours and then performed shape matching based on the template, while [33] used YOLOv3 and [34] combined U-Net with Mask R-CNN for vertebra localization without image preprocessing. In contrast, the study integrated image preprocessing with targeted cervical region extraction to amplify vertebral and foraminal features and to remove non-target areas before model training, thereby mitigating background interference. The results substantiated that this strategy localized C2–C7 more precisely and efficiently than prior methods.

In addition to providing accurate localization of vertebrae and neural structures, the system also incorporated automated measurement of intervertebral distances and distances between vertebrae and adjacent neural foramina. The measured distances showed correlations above 90% with neurosurgeon annotations, with the correlation of intervertebral distance reaching 97.5%, underscoring the high reliability of the system’s distance computation. These measurements facilitated longitudinal tracking of intervertebral and vertebra-to-neural foramen spacing served as valuable clinical indicators for assessing intervertebral disc degeneration, spinal stability, and potential nerve compression.

A current limitation is that pixel spacing was not considered, so the results were reported in pixels rather than physical units, precluding direct clinical length correspondence. Future work will calibrate pixel measurements to physical units (millimeter and centimeter), further optimize the model to improve accuracy, and extend the system to identify age-related spinal conditions such as osteophytes and intervertebral disc narrowing.

## 5. Conclusions

This study presented an automated cervical vertebra localization and distance measurement system capable of accurately detecting and identifying each vertebra in CSR images. Through image preprocessing, the structural features of each vertebra were significantly enhanced. By integrating image enhancement methods with the YOLOv8s model, the system achieved highly accurate identification of vertebral and neural positions. The recognition results were subsequently overlaid onto the CSR images, along with the computed intervertebral distances and the distances between each vertebra and the adjacent neural structures, thereby providing comprehensive visual information for clinical evaluation. This efficient and accurately automated system not only reduced manual annotation time and labor costs but also demonstrated strong potential for clinical diagnostics and telemedicine applications. The main contributions of this study are summarized as follows:

1. Increase in cervical spine localization accuracy:

In the preprocessing step of this study, cervical spine localization was performed to extract the cervical spine region for subsequent image enhancement and recognition. This method effectively eliminated irrelevant background and noise. The experimental results confirmed that, compared to the baseline accuracy of 93.30 without preprocessing, the proposed method significantly improved the accuracy to 99.50.

2. Highly accurate localization of C2 to C7 vertebrae:

By incorporating image preprocessing and enhancement methods, this study effectively accentuated the features of each vertebra, resulting in a substantial boost in localization performance. The proposed method achieved an outstanding overall vertebrae localization accuracy of 99.50, with even the anatomically challenging C2 and C7 vertebrae surpassing 99% accuracy. Notably, the accuracy for C7 localization improved by approximately 66.67% compared to existing methods, which only reached 60.00, marking a significant and noteworthy advancement in the field.

3. Automated positioning, labeling, and measurement system:

The system proposed in this study was based on YOLOv8s and was capable of automatically detecting and localizing vertebrae and neural structures, as well as measuring the intervertebral distances and the distances between vertebrae and neural structures. These measurements provided critical data for assessing spinal stability, intervertebral disc degeneration, and nerve compression. The system ensured consistent and accurate localization and measurement, while significantly reducing the need for manual annotation and data processing time.

In addition, this study introduced a user-friendly interface designed for healthcare professionals to facilitate the intuitive and practical application of the system in clinical settings. It was expected that this work would contribute to cervical spine healthcare by providing clinicians with an auxiliary tool to enhance workflow efficiency and improve patient care, ultimately benefiting both medical personnel and patients.

## Figures and Tables

**Table 1:** The hardware and software platforms in this study.

Hardware Platform	Version
CPU	11th Gen Intel(R) Core (TM) i7-100H
GPU	GeForce RTX 3070 Laptop GPU 8GB
DRAM	32GB DDR4 3200MHz
Software platform	Version
Operating System	Windows 11 Home 64-bit
Python IDE	PyCharm 2024.1

**Table 2:** The training data of Yolov8s.

	Train	Validation	Test
CSR	160	20	20

**Table 3:** Impact of cervical spine localization algorithm on vertebrae detection accuracy.

	Without Cervical Spinal Localization	This Work
Over All	93.30%	99.50%
C2	97.10%	99.30%
C3	91.40%	99.30%
C4	89.40%	99.40%
C5	100.00%	99.70%
C6	95.00%	99.60%
C7	87.00%	100.00%

**Table 4:** The K-Fold validation results for vertebra localization.

Fold	Accuracy (%)	Recall (%)	mAP50 (%)	mAP50–95 (%)
Fold1	99.10	97.70	99.30	80.80
Fold2	97.20	96.40	98.80	81.10
Fold3	97.70	96.50	98.10	80.10
Fold4	96.40	97.10	97.30	80.30
Fold5	99.60	99.60	99.50	81.90
Average	98.00	97.46	98.60	80.84

**Table 5:** The result of detection neural foraminal.

Class	Precision (%)	Recall (%)	mAP50 (%)
Neural Foraminal	97.35	95.70	97.70

**Table 7:** The PPMCCs of intervertebral distances and vertebra-to-neural foramen distances.

Case	PPMCC	
	Vertebra-to-Neural Foramen Distances	Intervertebral Distances
1	0.996	0.937
2	0.897	0.998
3	0.748	0.994
4	0.893	0.952
5	0.988	0.994
Average	0.904	0.975

## Data Availability

The datasets presented in this article are not readily available because they are part of an ongoing study and will be made available only after the completion of data collection and analysis. Requests to access the datasets should be directed to the corresponding authors at simonchi@mail.mcut.edu.tw or tsungychen@fcu.edu.tw.
